# The ethical landscape(s) of non-invasive prenatal testing in England, France and Germany: findings from a comparative literature review

**DOI:** 10.1038/s41431-021-00970-2

**Published:** 2021-10-04

**Authors:** Adeline Perrot, Ruth Horn

**Affiliations:** The Ethox Centre, Nuffield Department of Population Health, University of Oxford, Oxford, UK

**Keywords:** Ethics, Genomics

## Abstract

Since 2019, England, France and Germany have started offering NIPT as a publicly funded second-tier test for common chromosomal aneuploidies (trisomy 21, 18 and/or 13). Despite these benefits, the introduction of NIPT into routine prenatal care also raises a number of ethical concerns. In this paper, we analyse how these issues are discussed differently across countries, echoing the different socio-political particularities and value-systems that shape the use and regulation of NIPT in a specific country. The international comparison between England, France and Germany shows how each country defines the principle of reproductive autonomy and weighs it against other principles and values, such as, human dignity, disability rights and the duty of care of health professionals. In terms of methodology, our literature review focuses on arguments and regulations of prenatal testing and reproductive choices (specifically on NIPT), through the investigation of regulatory, parliamentary, scientific, medical, association, institutional and media sources. The comparative review helps to better understand ethical questions discussed with regard to NIPT, and, more broadly, to prenatal genomic testing, and the limits associated with reproductive autonomy in the three countries studied. Whereas reproductive autonomy is valued in each country, it is understood and implemented differently depending on the socio-cultural context, and on what other principles are evoked and how they are defined.

## Introduction

Non-invasive prenatal testing (NIPT), also known as Cell-free fetal (cff) DNA screening, is a rapidly developing technology that is constantly widening its scope and offering new approaches to fetal medicine. According to some authors, NIPT is at the ‘vanguard of genomic medicine’ [1].

Since 2011, NIPT has been commercially available in over 60 countries [2]. Recently, a number of countries such as England, France and Germany, have started offering NIPT as a publicly funded second-tier test for common chromosomal aneuploidies (trisomy 21, 18 and/or 13). NIPT has a number of advantages over the conventional combined first‐trimester screening (cFTS) consisting of ultrasound examination and serum markers [3]; it can be performed earlier in pregnancy (from 9 to 10 weeks), is considered more accurate in detecting common chromosomal aneuploidies and with a lower false positive rate [4], and it decreases the number of invasive tests that carry a low risk of miscarriage.

Despite these benefits, the introduction of NIPT into routine prenatal care also raises a number of ethical concerns about, for example, the risk of routinisation undermining reproductive choices or increasing discrimination against children living with a trisomy. These, and other issues, are discussed differently across countries, echoing different socio-political particularities and value-systems that shape the use and regulation of NIPT in a specific country. Analysing the differences between countries and their particularities will contribute to a better understanding of the ethical issues at stake.

In this paper, we compare England, France and Germany, three European countries that offer NIPT as a second-tier test, but define the threshold for giving free access to NIPT differently, echoing their different public health priorities. We will show how the public discourses about prenatal testing, screening policies, professional regulations and relevant laws, in the three countries reflect their different value-systems. The comparison shows how each country defines the principle of reproductive autonomy and weighs it against other principles and values such as human dignity, disability rights and the duty of care of health professionals. Our analysis sheds new light on different meanings of reproductive autonomy within various socio-cultural contexts and how this impacts on the use and regulations around prenatal genetic testing. Such an investigation is an important step in advancing the ethical and policy debates regarding NIPT in different countries.

## Methods

Our study provides an insight into the similarities and differences between England, France and Germany through a comprehensive literature review focusing on arguments about, and regulations of, prenatal testing and reproductive choices (specifically on NIPT).

Between December 2020 and April 2021, we reviewed approximately 250 sources in legal and regulatory texts; public reports of national ethics committees and professional bodies; parliamentary debates; medical press; academic literature in prenatal genetics, bioethics, social sciences; and daily press. The sources covered a wide range of issues related to the implementation of NIPT such as ethical and practical issues; public, political and scientific debates; regulations and guidelines; criteria for offering the test free of charge; and communication of results. We focused on literature regarding the English, French and German context, since 2011, when NIPT became first available in the private sector before several countries decided to fund it within their public health system. We searched the databases of Cairn journals (Humanities and Social Sciences), Google Scholar, PubMed and SAGE journals. The review of documents and terminologies was guided by the following questions: how is NIPT regulated in each country? Who are the main stakeholders and institutions involved in the offer of NIPT? What are the ethical arguments used by stakeholders in public, political, parliamentary and professional debates? What are the meanings of these arguments in each context? We identified differences with regard to the ethical questions associated with NIPT and the ways these are discussed in each country.

This analysis is part of a wider comparative study combining literature review, empirical research (semi-structured interviews with stakeholders) and conceptual analysis to explore the ethical issues arising from prenatal genetics in England, France and Germany [5].

### Implementation of NIPT in antenatal care: regulations and public policies

We propose to start with an overview of the practical regulations defining how and to whom NIPT is (or will) be offered as a publicly funded screening test in the three countries studied. In France, since January 2019, NIPT is reimbursed as a second-tier screening test, between the 11 and 14th week for pregnancies with a probability of trisomy 21 (T21) (between 1:51 and 1:1000), following cFTS (UK National Screening Committee (UK NSC)). In England, in 2016, UK NSC has recommended that NIPT should be offered to women with an increased probability of having a baby with T21, T13 and T18 with a higher cut-off than in France, at 1:150, following first trimester combined (11–14 weeks) or second trimester quadruple screening (14–20 weeks). This recommendation was implemented in June 2021 as part of the NHS Fetal Anomaly Screening Programme (FASP). Similarly, in Germany, it was decided in 2019 to cover NIPT for pregnancies with an increased likelihood of T21, T13, and T18 through the publicly funded health insurance system from 2021. Unlike in France and in England, the risk cut-off is determined individually and is independent of a quantifiable risk calculation [6, 7]. In Germany, a statistically increased risk is not seen as a sufficient criterion to reimburse the test [8]. It is stated that only a particular situation, where the pregnancy and its consequences present a burden to the pregnant woman and could lead to serious harm of her mental health, can justify the test; hence offering the test requires a case-by-case decision [6].

In the three countries, pregnant women are usually referred by their gynaecologist–obstetrician, midwife or general practitioner to fetal medicine units, prenatal clinics or medical genetics service that offer NIPT. Because of the high accuracy of NIPT in detecting the common chromosomal trisomies (T21, T13 and T18), and in particular for T21, all three countries have decided to publicly fund the test only for these trisomies, and not for other conditions or traits for which the test is less accurate.^1^

NIPT for T21, T18 and T13 is not used as a diagnostic test at present, and so a positive NIPT test requires further testing for confirmation in the second trimester (e.g. amniocentesis or chorionic villus sampling (CVS)) [9]. However, because of its higher accuracy than cFTS in detecting trisomies, fewer women will need to undergo such confirmatory tests, which may involve some, albeit minimal, risk of miscarriage for amniocentesis and CVS (0.11% and 0.22% respectively) [10]. This clinical advantage has been one the main arguments mobilised for its use in routine prenatal care in the three countries, but also in other countries where NIPT is implemented.

### Similarities in the ethical concerns across the three countries

Despite the advantages of the test, offering NIPT as part of routine clinical service raises important ethical questions. Through our literature review, we identified similarities among issues raised in England, France and Germany, for example by patient associations, national ethics committees, medical experts, politicians and journalists. The issues presented here are also largely discussed in the international literature on NIPT and are not particular to the three countries. For example, while it is acknowledged that NIPT offers a range of benefits such as earlier and more accurate results compared to cFTS, better understanding of fetal aneuploidies and, ultimately, better informed reproductive decision-making, concerns are expressed that its use in routine antenatal care may increase the risk of stigmatisation and discrimination of people living with an autosomal aneuploidy, having a negative impact on the support for women who decide to raise a child with a trisomy [11–13].

One other major concern discussed in the literature is that NIPT could become a routine test, which could intensify the already existing challenge of prenatal testing, potentially putting pressure on women to undergo testing, and hence undermining informed decision-making, and weakening reproductive autonomy [14]. Other arguments concern the risks of prenatal sex-selection and the risks of screening for ‘less severe’ conditions, adult-onset conditions and carrier status [15, 16]. Especially in England, there are concerns about prenatal sex-selection as expressed in parliamentary questions addressed to the Department of Health in 2016, and in the Labour Party’s call to ban early fetal sex testing in 2018 (*BBC*, Labour calls for ban on early foetus sex test, 2018). There are also ethical questions about the communication of results and information management in cases of misattributed paternity, secondary or incidental findings that may have implications not only for the fetus’ health but also the health of the mother or other family members [17]. Furthermore, where women use commercial companies, the quality of information returned and the counselling provided by some of these companies is criticised and challenges the idea of informed decision-making [18]. However, ethical issues of direct-to-consumer testing are not the focus of this paper.

### Different perspectives on reproductive autonomy

Through our comparison, differences emerged in the ways in which the ethical issues related to reproductive autonomy are addressed in each country. First of all, in England, the debate highlights the risk that NIPT could be recommended to women as a standard test, a ‘simple blood test’ that may not involve the same level of pre-test counselling and information as an amniocentesis or CVS. The concern, which is particularly highlighted in a 2017 report of the Nuffield Council on Bioethics is, that the ‘less invasive nature’ of NIPT could make it difficult for women to refuse the test [19] and, therefore, undermine informed consent and reproductive autonomy [19, 20]. In order to address this concern, there is a strong focus on understanding and recognising the needs, beliefs and preferences of women in order to enable them to make autonomous decisions. In 2020, a collective of professional bodies (Royal College of Obstetricians and Gynaecologists, Royal College of Midwives, Society and College of Radiographers) have developed a consensus statement suggesting that information about NIPT is provided by healthcare professionals in a non-directive way so that women are able to make choices that are right for them. In the same way, the Public Health England Fetal anomaly screening programme, the Nuffield Council on Bioethics and NHS England emphasise clarity, accuracy and non-directiveness when informing women on benefits and limitations of the NIPT [19, 21]. Parliamentary questions^2^ and daily press^3^ also reflect the importance of helping women to make informed decisions by providing appropriate information, explaining the different options, offering support and respecting their decision. This reflects the autonomy-focused approach in England [22].

Concerns are raised also in France about the negative impact NIPT may have on women’s choices, emphasised in a regulatory framework of 2018 stating that: ‘The woman is at the centre of the system and makes all decisions regarding her pregnancy. Her autonomy must be respected’ [23]. However, unlike England, France puts emphasis on the content of the information (e.g. organisation of screening and timeframes, results communication) rather than on it being non-directive. The focus is less on enabling women to make informed decisions than on the need to protect women from the risks of ‘commercial exploitation’ of genetic screening tests (NIPT) and ‘leaving couples alone and helpless when faced with non-validated tests’ [13], as described by the National Ethics Committee (Comité Consultatif National d’Ethique), in 2013. This concern about women as victims of the commercialisation of ‘risk’, the ‘lobby of diagnostic merchants’ and ‘pregnancy monitoring’ is present also in the daily press^4^. According to these accounts, women are described as likely to lack full understanding of complex genetic information and therefore ought to be protected in order to make their own decisions. Consistent with this discourse, empirical studies have confirmed a certain paternalistic attitude among French health professionals when it comes to prenatal decisions and informing women of the choice they have with regard to prenatal testing [24]. In France, the possibility for women to make informed choice is important [13] and has been included in the law (Loi n° 2002–303 du 4 mars 2002), reflecting an increased focus on a patient-centred approach as part of what is called a ‘health democracy’ (démocratie sanitaire) [25, 26]. In the case of NIPT, however, the concern for women’s autonomous choice shows itself in the form of a rather protective approach that can be seen as restricting reproductive choice. In contrast, although France insists on free choice and the right to revoke the decision of consent to carry out examinations [23], it puts more emphasis on the content of the information than on the way it is delivered (provide fair and appropriate information, inform about stages of screening and diagnosis, times between the different examinations, distinction between “risk” and certainty of diagnosis, possibility to continue or not the pregnancy, etc.).

In Germany, the debate on reproductive autonomy in the context of NIPT often focuses on the woman’s right not to know’ or her right to decline available prenatal tests [27–29]. It is also suggested that, in addition to information provided by professionals, a woman should be put in contact with associations or families who have a child living with a trisomy [6, 30], so, she is able to make a fully informed decision about whether to continue or terminate her pregnancy. Furthermore, concerns are raised about the scope and limit of the respect for the dignity of the future child, and its ‘right to life’ and to be recognised as a ‘human being’. The potential conflict between the future child’s ‘right to life’ and the woman’s right over her own body was also highlighted in the 2019 report of the Committee on Education, Research and Technology Assessment of the German Bundestag, on the current situation and development of prenatal diagnosis [31]. Generally, in the German debate, we notice a strong focus on enabling women to make their own informed decisions as well as a cautious approach with regard to new reproductive technologies that could compromise the dignity of human life from its very beginning. Public debates on bioethical issues in post-war Germany often emphasise the importance of respecting both individual autonomy and human dignity [32], two principles that echo Kant’s influence on contemporary debates. Policies aim to weigh the autonomy and dignity of one human life, that of the woman, against the autonomy and dignity of another human life, the future child. The first (woman’s autonomy and dignity) can outweigh the latter (future child’s autonomy and dignity) only where there is guarantee that the woman makes a truly informed autonomous decision free of any undue pressure [27].

We have seen how reproductive autonomy is discussed differently in each country, and how it is associated with different ethical concerns, echoing different prevailing norms and values in each country. These aspects and nuances are also reflected in the various public reactions in England, France and Germany, as we will see below.

### Public reactions and mobilisations

The incorporation of NIPT into public healthcare systems has led to criticism at different levels depending on perceptions and values associated with disability, new biomedical technologies and reproductive rights. In England, several campaigns have put NIPT on the political agenda. For example, in 2016, the ‘Don’t Screen Us Out’ campaign, led by a Down’s syndrome advocacy group, started a petition which was signed by over 900 people with Down’s Syndrome and their families denouncing the violation of the Convention on the Rights of Persons with Disabilities (CRPD). That same year, a documentary by the British actress, Sally Phillips, ‘A World Without Down’s Syndrome?’ (BBC, 5 October 2016) denounced the ‘devaluation’ of families and children living with Down’s syndrome, the ‘biased information’ given to pregnant women about the condition, and the risk of the ‘decline’ of members of this community. However, in England, such critical voices are outweighed by the value accorded to the right of women to make independent and informed choices [33, 34]. Furthermore, as evidenced in parliamentary questions^5^ and the daily press,^6^ the public discourse favours the benefits and technological progress of NIPT such as its safety and accuracy.

In Germany, protests by civil society organisations (German Down Syndrome InfoCenter, KIDS Hamburg, Lebenshilfe, Joint Declaration on World Down Syndrome Day, Network against Selection through Prenatal Diagnostics) emerged in 2011, following an investigation by the weekly newspaper *Die Zeit*, revealing that the laboratory LifeCodexx had received around 300,000 Euros from the Federal Ministry for Research and Education for test development of NIPT. In response to these protests, the Federal Government commissioned the German Ethics Council to provide an expert opinion on NIPT [35]. The report mainly focuses on arguments in favour or against the public funding of NIPT. It offers an insight into the German context where access to NIPT through individualised genetic counselling is prioritised over the definition of a numerical risk threshold. Following a call for political transparency and a public debate around biotechnological innovations through an Open letter to the Federal Joint Commission (G-BA) by several patient organisations in 2016, there has been a plea for regular evaluations of the new prenatal test, including its ethical and social implications. In Germany, despite the importance given to reproductive autonomy, public attitudes showcase scepticism towards prenatal genetic technologies [36, 37]. As mentioned above, we notice also here a certain criticism and questioning of the use of new technologies, and an emphasis on risk prevention and management in the German debate [37]. These public attitudes reflect on the implementation of prenatal technologies in the public healthcare system; for example, cFTS is not reimbursed if there is no reason for concern such as advanced maternal age, which explains the slightly lower uptake of prenatal screening when compared to other European countries [38].

In France, criticism of NIPT has emerged in different formats within the associative space: examples of this are the organisation of a conference by the Jérôme Lejeune Foundation, ‘Stop Discriminating Down’, in 2017, aiming to denounce ‘the mass elimination of children with Down’s syndrome before birth’ by the arrival of NIPT; or the publication of the book, ‘Les premières victimes du transhumanisme. Dépistage prénatal de la trisomie 21’ (The first victims of transhumanism. Prenatal screening for Down’s syndrome), by Jean-Marie Le Méné, president of the Jérôme Lejeune Foundation, in reaction to the reimbursement of the test in 2019. Also, a ‘March for Life’ was organised in 2019, in Paris, to warn against ‘a new step in eugenics’ as a response to the decision to reimburse NIPT. In France, these public reactions were largely driven by associations and had no impact on policies.

In addition to these critical reactions to NIPT, in the following section, we will discuss how the charges of ‘eugenics’ have emerged at the centre of public debates in France and Germany, while remaining relatively marginal in England.

### Charges of eugenics as a means of denouncing NIPT

In the three countries, the reference to the risk of a drift toward ‘eugenics’ appears in different forms and is used as an argument, especially in France and Germany, against the implementation of NIPT. While advocates of NIPT emphasise that the use of the test relies on an individual choice [33, 39] and is not linked to eugenic or coercive policy [40], critics express concerns that individual ‘abortion’ practices could lead to widespread selection of babies and the lack of tolerance and care for people with disability [41].

In England, concerns about the risk to ‘screen out’ and, hence, significantly reduce the number of births of children with Down’s syndrome have been expressed, among others, by the campaign group ‘Don’t screen us out’, the Church of England,^7^ and in parliamentary questions.^8^ Yet, generally, the public debate in England focuses more on the offer of NIPT and how to guarantee respect for women’s decisions, accuracy of information, regular training of health professionals, and designing care pathways for women continuing their pregnancy with a baby who has Down’s syndrome [42]. Also the Church of England states that it ‘welcomes medical advances’ as long as women receive ‘comprehensive and unbiased information about the condition’ [43]. It is rare in England to hear strong accusations against NIPT as a form of ‘eugenics’.

In France, the association between NIPT and ‘eugenics’, ‘elimination’ ‘eradication’ or ‘selection’ is more explicit and recurrent. In 2007, prior to the revision of the bioethics laws, *Le Monde* published an interview with the president of the National Consultative Ethics Committee, Didier Sicard, where he warned against the risk of eugenics and ‘social eradication’ if prenatal screening becomes routinised. This perspective is also reflected in parliamentary debates on NIPT where representatives of the centre-right refer to ‘eugenics’^9^ or ‘elimination’.^10^ Similarly, the risk of increasing fetal selection, a ‘new form of eugenics’ (no longer through the State, but the individual) and of further discrimination, is highlighted by the National Consultative Ethics Committee in its 2016 report n° 124. The question of the re-emergence of ‘eugenics’ with reference to Nazism is also raised by established media,^11^ the Catholic Church in Paris which denounces the pressure on women to be screened for Down’s syndrome [44] and the Jérôme Lejeune Foundation which uses the terms ‘elimination’ [45], ‘disappearance’, ‘extinction’ [46] and ‘eradication’ [47] to refer to NIPT. Despite the links made between NIPT and ‘eugenics’ in the French debate, there is no evidence that expecting parents would desire a ‘perfect child’ rather than just wanting to bring a healthy child into the world [40]. It should therefore be pointed out that there is a fine line between health and expectation of normality and that this should not lead to a search for the improvement of the genetic characteristics of the child, in the sense that we understand the term ‘eugenics’ here [48]. In Germany, despite its Nazi past, ‘eugenics’ is not as explicitly referred to as in France. The arguments used against potential discrimination or genetic selection, as a consequence of NIPT, are based on the principle of respect for the ‘dignity of the unborn child’ [27, 31], which may conflict with the respect for the woman’s dignity as an autonomous individual [27, 28]. However, the possibility to terminate pregnancy in order to protect the life or the mental or physical health of the woman (Strafgesetzbuch § 218a) indicated that the dignity of the unborn child can be suspended in favour of the protection of the dignity of the pregnant woman. As mentioned above, the German discourse is inspired by the Kantian concept of ‘human dignity’, and its adoption as the first constitutional principle since 1949.

As prenatal technologies such as NIPT have evolved, all three countries have seen discussions about the potential risk of ‘eugenics’. Although each country emphasises that reproductive decisions belong to the woman, critical voices caution against decisions that could be implicit forms of ‘eugenics’. To date however, there is no evidence that the introduction of NIPT has led to an increase in termination rates due to fetal anomalies [49]. The results highlight that, in many cases, women accept NIPT to obtain information about the fetus’ health and prepare for the child’s arrival without intending to terminate pregnancy if an aneuploidy is discovered [49].

## Conclusion

The comparison between the English, French and German discourses shows similarities and differences with regard to the values and principles that are at play in each country’s debate on NIPT, and how they relate to various socio-cultural contexts. Although reproductive autonomy is valued in each country, it is understood and implemented differently: with a strong focus on informed consent and choice in England, a focus on medical information and protection in France and a focus on the balance between the ‘right to know‘ and ‘not to know’ in Germany. These differences depend on the socio-cultural context and on what other principles (e.g. disability rights, human dignity, or professional duties to promote health and reduce technology-related risks) are evoked, and how they are defined and weighed against each other.

Our comparative literature review allowed us to highlight the influences, values and principles that shape national debates and policies. To further unpack these values and principles, and better understand the arguments and policies regarding NIPT in each country, further qualitative research is required to investigate various stakeholders’ (women, health professionals, scientists and policy-makers) positions and motivations. Such an in-depth understanding will contribute to both the ethical and societal debates on NIPT, and feed into policy considerations of best practice regarding the delivery of NIPT, and other new prenatal genetic technologies, in the three countries.

## Data Availability

Data sharing not applicable to this article as no datasets were generated or analysed during the current study.

## Footnotes

1 Other than those involving the chromosomes 13, 18, 21, X and Y. 9.Scott F, Bonifacio M, Sandow R, Ellis K, Smet ME, McLennan A. Rare autosomal trisomies: Important and not so rare. Prenatal Diagnosis. 2018;38(10):765–71.

2 UK Parliament. Pregnancy: Screening. Department of Health written question—answered on 23rd March 2016. 2016; UK Parliament. Down’s Syndrome: Abortion. Women and Equalities written question—answered on 18th February 2019.

3 Newsom A. New test for Down’s raises important ethical questions. *The Times*. 2011; Turner J. A better test for Down’s shouldn’t scare us. Parents must have the right to choose screening but that doesn’t mean we don’t cherish people with the syndrome. *The Times*. 2016.

4 Collectif. Le dépistage sanguin prénatal, une avancée en trompe-l’œil. *Le Monde*. 2018; Collectif. Dépistage prénatal: Les marchands de risques. *Le Monde*. 2009.

5 UK Parliament. Down’s Syndrome. Department of Health written question—answered on 4th April 2016. 2016; UK Parliament. Pregnancy: Screening. Department of Health written question—answered on 22nd November 2016.

6 A risk free blood test for Down’s syndrome in pregnancy. Potential advances in prenatal screening. *The Times*. 2008; Rayment T. Blood test for Down’s will cut miscarriages. The use of amniotic needles, that have caused thousands of miscarriages, could be consigned to history thanks to the new test. *The Sunday Times*. 2011; Devlin H. Fears over new Down’s syndrome test may have been exaggerated, warns expert. *Guardian*. 2016.

7 General Synod. Valuing People with Down’s Syndrome. 2018.

8 UK Parliament. Down’s Syndrome Department of Health written question—answered on 23rd March 2016; UK Parliament. Down’s Syndrome: Screening. Department of Health and Social Care written question—answered on 23rd September 2020; UK Parliament. Down’s Syndrome Department of Health and Social Care written question—answered on 11th February 2019.

9 Assemblée Nationale. Question N° 30088 de Mme Véronique Besse (Non inscrit—Vendée). 2013; Assemblée Nationale. Question N° 43050 de M. Jean-Pierre Decool (Union pour un Mouvement Populaire—Nord). 2013.

10 Assemblée Nationale. Question N° 43818 De Mme Véronique Besse (Non inscrit—Vendée). 2013; Assemblée Nationale. Question N° 43817 de M. Rudy Salles (Union des démocrates et indépendants—Alpes-Maritimes). 2013; Assemblée Nationale. Question N° 44822 de M. Philippe Gosselin (Union pour un Mouvement Populaire—Manche). 2013.

11 Perez M. L’enfant parfait ou la tentation de l’eugénisme. L’Analyse de Martine Perez, chef de service à la rubrique Sciences du *Figaro*. 2008; Alberganti M. Le diagnostic prénatal engendre-t-il une nouvelle forme d’eugénisme? *France Culture*. 2012. Podcast.
